# Knowledge and Attitudes of General Practitioners and Sexual Health Care Professionals Regarding Human Papillomavirus Vaccination for Young Men Who Have Sex with Men

**DOI:** 10.3390/ijerph15010151

**Published:** 2018-01-18

**Authors:** Samuel W. D. Merriel, Carrie Flannagan, Joanna M. Kesten, Gilla K. Shapiro, Tom Nadarzynski, Gillian Prue

**Affiliations:** 1 Centre for Academic Primary Care, Bristol Medical School, University of Bristol, Bristol BS8 2PS, UK; 2 Institute of Nursing and Health Research, Londonderry BT52 1SA, UK; c.flannagan@ulster.ac.uk; 3 Bristol Medical School, University of Bristol, Bristol BS8 2PS, UK; jo.kesten@bristol.ac.uk; 4 The National Institute for Health Research Health Protection Research Unit in Evaluation of Interventions, Bristol Medical School, University of Bristol, Bristol BS8 2PS, UK; 5 The National Institute for Health Research Collaboration for Leadership in Applied Health Research and Care West (NIHR CLAHRC West), University Hospitals Bristol NHS Foundation Trust, Bristol BS1 2NT, UK; 6 Department of Psychology, McGill University, Montreal, QC H3A 1A2, Canada; gilla.shapiro@mail.mcgill.ca; 7 Department of Psychology, University of Southampton, Southampton SO17 1BJ, UK; t.nadarzynski@soton.ac.uk; 8 School of Nursing and Midwifery, Queens University, Belfast BT7 1NN, UK; g.prue@qub.ac.uk

**Keywords:** vaccine uptake, vaccine communication, sexual minorities, papillomaviruses

## Abstract

Men who have sex with men (MSM) may be at higher risk for human papillomavirus (HPV)-associated cancers. Healthcare professionals’ recommendations can affect HPV vaccination uptake. Since 2016, MSM up to 45 years have been offered HPV vaccination at genitourinary medicine (GUM) clinics in a pilot programme, and primary care was recommended as a setting for opportunistic vaccination. Vaccination prior to potential exposure to the virus (i.e., sexual debut) is likely to be most efficacious, therefore a focus on young MSM (YMSM) is important. This study aimed to explore and compare the knowledge and attitudes of UK General Practitioners (GPs) and sexual healthcare professionals (SHCPs) regarding HPV vaccination for YMSM (age 16–24). A cross-sectional study using an online questionnaire examined 38 GPs and 49 SHCPs, including 59 (67.82%) females with a mean age of 40.71 years. Twenty-two participants (20 SHCPs, *p* < 0.001) had vaccinated a YMSM patient against HPV. GPs lack of time (25/38, 65.79%) and SHCP staff availability (27/49, 55.10%) were the main reported factors preventing YMSM HPV vaccination. GPs were less likely than SHCPs to believe there was sufficient evidence for vaccinating YMSM (OR = 0.02, 95% CI = 0.01, 0.47); less likely to have skills to identify YMSM who may benefit from vaccination (OR = 0.03, 95% CI = 0.01, 0.15); and less confident recommending YMSM vaccination (OR = 0.01, 95% CI = 0.00, 0.01). GPs appear to have different knowledge, attitudes, and skills regarding YMSM HPV vaccination when compared to SHCPs.

## 1. Introduction

Human papillomavirus (HPV) vaccination of young men who have sex with men (YMSM) (age 16–24) potentially has important implications for cancer prevention worldwide. HPV is one of the most common sexually transmitted infections [1]. Over 70% of MSM are carriers of HPV [2,3]. HPV infection is associated with other anogenital and oropharyngeal cancers [4]. Anal cancer incidence has increased rapidly in recent years [5], with approximately 95% of anal cancers caused by HPV [6]. MSM (men who have sex with men) carry a disproportionate burden of anal cancer (15:1 compared with heterosexual men) [7]. Relative to human immunodeficiency virus (HIV)-negative men or women, HIV-negative MSM have a 4-fold higher risk of developing anal cancer, and HIV-positive MSM have up to an 80-fold higher risk [8]. Approximately 72% of oropharyngeal cancer cases in the United States from 2008–2012 was attributable to HPV, with an annual incidence rate of 7.6 per 100,000 population [9].

Prevention of HPV-related disease is a key public health issue. The United Kingdom’s (UK) current strategy is to offer publicly funded vaccination only to girls aged 12–14 (prior to the legal age of consent at 16 years), which is intended to protect males through herd immunity. This decision was made on the basis of cost effectiveness [10], although more recent studies have called this into question [11]. This benefit does not extend to MSM. Public Health England estimates that 3.2% of the UK population are lesbian, gay, or bisexual [12], which suggests that almost one million UK men may not protected from HPV-associated anogenital warts and cancers. In November 2015, the Joint Committee on Vaccination and Immunisation (JCVI) recommended the HPV vaccination programme be extended to MSM aged up to 45 years via genitourinary medicine (GUM) clinics, HIV clinics, or opportunistically through general practice clinics (GPs) [13]. 

Vaccination is likely to be most efficacious before exposure to HPV [14], however the majority of men do not identify as gay or bisexual before they engage in sexual contact with other men [15], and many men do not disclose their sexual identity and/or behaviour to their physician. In attempts to address this issue, UK healthcare professionals (HCP) have recently been issued guidance from NHS England recommending that they enquire about a patient’s sexual orientation at “every face to face contact with the patient, where no record of this data already exists” [16]. Such policies have the potential to exacerbate stigmatisation of LGBTQ patients accessing healthcare services if they feel they will be asked to disclose their sexual orientation every time they access a service, whether it is relevant to their presenting complaint or not [17]. Best practice guidance for discussing sexual behaviour has been produced from UK charities, such as Stonewall [18].

It is crucial to engage widely with HCPs expected to vaccinate YMSM against HPV. Patients and parents of younger children place a strong emphasis on the recommendations (or otherwise) of a HCP in decision-making regarding vaccinations [19,20,21]. GPs will arguably have more opportunity to vaccinate men before sexual debut compared to GUM clinics, given that men are more likely to attend a GUM clinic after the first sexual encounter [15]. It is also important to identify appropriate strategies to support any new HPV vaccination programmes in the future and highlight any barriers and facilitators to the programmes’ effective implementation.

In a survey of 131 sexual healthcare professionals (SHCPs), 95% of clinicians supported a targeted HPV vaccination programme in MSM within GUM services but expressed concern that alone this strategy was too late and too limited for most MSM [22]. This study was specific to clinicians with expertise in sexual health, and did not include other HCPs who may be involved in vaccination, such as GPs. It was also conducted prior to the recent JCVI recommendation. The aim of this study is to understand and compare the knowledge, perceptions, and attitudes of UK GPs and SHCPs regarding HPV vaccination for YMSM.

## 2. Materials and Methods

An exploratory cross-sectional survey of GPs and SHCPs was conducted as part of a mixed-methods study. SCHPs included GUM consultants, doctors-in-training, and nurses working in sexual health clinics. Between September 2016 and January 2017, convenience sampling was used to recruit participants through an email invitation distributed by the Royal College of General Practitioners (RCGP), British HIV Association (BHIVA), British Association for Sexual Health and HIV (BASHH), Society of Sexual Health Advisors (SSHV), Society for the Study of Sexually Transmitted Diseases in Ireland (SSSTDI), and the University of Bristol Centre for Academic Primary Care (CAPC). The email invitation included a link to the online survey and a participant information sheet which explained that participation implied consent. At the end of the anonymous survey, GPs were invited to provide contact details if they wished to take part in a follow-up interview (findings not yet published). Similar to Nadarzynski et al. [23], participants were asked to distribute the e-survey to co-workers to increase the number of responses using snowballing techniques.

The questionnaire aimed to capture knowledge and attitudes towards HPV vaccination for YMSM, as well as any barriers or facilitators. YMSM were chosen as the focus for this study as greater understanding of factors affecting HPV vaccination in this age group could improve the efficacy of HPV vaccination programmes for MSM by targeting younger men before they engage in sexual activity. Questionnaire content was informed by a study steering group comprising two lesbian, gay, bisexual, transgender and queer (LGBTQ) group stakeholders and three MSM sexual health researchers from England and Northern Ireland, and piloted with HCPs prior to wider distribution. The questionnaire was adapted from a HCP HPV attitude scale developed by Nadarzynski et al. [23], and a HCP pre-exposure prophylaxis (PrEP) knowledge and attitude scale [24]. Question items focused on the barriers and facilitators to vaccinating YMSM provided pre-specified options based on existing literature, with an option for free text responses. Individual questionnaire items used either binary (“yes” or “no”) or ordinal (“high”, “medium”, “low”) response measures for knowledge questions. Basic demographic information, including participant age, gender, clinical role, and years of experience were gathered.

Descriptive statistics summarised demographic, attitude, and knowledge data. Fisher’s exact testing and unpaired *t*-tests were utilised for comparison of demographics. Due to null responses for some categories, sexual orientation was converted to a binary variable (“heterosexual or straight” vs. “gay, lesbian, or bisexual”) for the analysis. Ordinal knowledge variables were converted to binary responses (“high/medium” vs. “low/none”). Attitudinal responses were converted into positive (“yes”) or negative (“unsure” or “no”) binary variables. Simple and multiple logistic regression techniques were utilised to compare the responses of GPs and SHCPs to the knowledge and attitude questions. Adjusted analysis controlled for the effects of participant age, gender, sexual orientation, and years of experience. Hosmer–Lemeshow goodness of fit testing was performed to assess accuracy of multiple logistic regression models. All analysis was conducted using Stata version 14 (StataCorp. 2015. Stata Statistical Software: Release 14. College Station, TX, USA: StataCorp LP.).

### Ethical Approval

This study received ethical approval from the Queens University (Belfast) School of Nursing and Midwifery Research Ethics Committee (ref. 39.GPrue.05.16.M8.V2). 

## 3. Results

In total, 87 participants completed the survey. Demographic data was incomplete for three SHCPs, but overall individual question response rates were high (range: 94.25–100%). Thirty-eight GPs and 49 SHCPs (35 GUM specialists, 8 specialist nurses, 3 hospital sexual health specialists, and 3 other) completed the questionnaire. Participants included 59 females (67.82%), with a mean age of 40.71 years, and a median 14 years of experience (IQR (Interquartile Range) 8, 24). There were no significant differences between GPs and SHCPs. Further demographics are shown in Table 1.

SHCPS were more likely than GPs to have vaccinated a YMSM patient against HPV (20/49 (47.83%) vs. 2/38 (5.6%), *p* < 0.001), more likely to be aware of the recent JCVI recommendations (adjusted OR = 0.03, 95% CI = 0.01, 0.11), and more likely to report they knew enough to have an informed discussion with MSM about HPV vaccination (adjusted OR = 0.04, 95% CI = 0.01, 0.14). Thirty GPs (78.95%) stated they have a “low level of knowledge” or “no knowledge” of HPV vaccination for YMSM, compared to 6 (12.24%) SHCPs (adjusted OR = 0.02, 95% CI = 0.00, 0.10), but there were no significant differences in knowledge ratings regarding overall HPV knowledge or HPV in females (see Table 2). 

GPs attitudes towards HPV vaccination in YMSM differed from SHCPs (see Table 3). GPs were less likely to agree that HPV vaccination should be widely available for both genders (adjusted OR = 0.30, 95% CI = 0.09, 0.98) or MSM (adjusted OR = 0.30, 95% CI = 0.09, 0.98) based on current evidence. Even if a gender-neutral programme existed in the UK, GPs were less likely to recommend HPV vaccination to MSM (adjusted OR = 0.33, 95% CI = 0.13, 0.88) and they were less likely to believe the majority of YMSM would be willing to receive the vaccine (adjusted OR = 0.13, 95% CI = 0.04, 0.41). Paradoxically, there was no significant difference in the numbers of GPs who would recommend the HPV vaccination to their own son (33/38, 86.84%) compared to SHCPs (49/49, 100%, *p* = 0.36).

When asked about whether they ask patients about sexual orientation “if it is relevant to the consultation”, there was no difference between the responses of GPs (31/38, 81.58%) and SHCPs (40/46, 86.96%, *p* = 0.57). GPs were much less likely to believe that a young person would disclose their sexual orientation to them (adjusted OR = 0.17, 95% CI = 0.06, 0.50), less confident that they had the skills to identify YMSM who may benefit from HPV vaccination (adjusted OR = 0.03, 95% CI = 0.01, 0.15), and they reported lower levels of confidence in recommending HPV vaccination for YMSM (adjusted OR = 0.04, 95% CI = 0.01, 0.18).

GPs and SCHPs reported different factors that would most affect their ability to deliver HPV vaccination for young MSM (see Table 4). GPs highlighted “no time” as a key limiting factor (25/38, 65.79%), while SCHPs felt “staff availability” (27/49, 55.10%) was the most important limitation. The majority of GPs (28/38, 73.68%) felt that additional training was needed to support HPV vaccination for MSM in primary care, while SHCPs felt computer prompts would be most useful (18/35, 51.43%) (see Table 5).

## 4. Discussion

This is the first UK-based study to examine the knowledge, perceptions, and attitudes of GPs and SHCPs since the JCVI updated its recommendations to include offering HPV vaccination for MSM for men under 45 years opportunistically in GUM and primary care. The survey findings suggest that compared to SHCPs, GPs were less aware of the evidence for HPV vaccination for MSM, and reported less confidence in recommending HPV vaccination to YMSM. GPs felt that lack of time and training were the main barriers to HPV vaccination for YMSM, whereas SHCPs had greater concerns about vaccine availability.

A similar survey targeting SHCPs was conducted prior to the JCVI recommendation of a targeted vaccination programme for MSM [23]. SHCP attitudes around perceived value, health behaviours, and capabilities are consistent across the two studies, and there are no clear changes following the JCVI recommendation. This is probably not surprising given their clinical interest in preventing the spread of HPV and exposure to MSM with sexual health problems in clinical practice. Interestingly, 74% of respondents in that study “agreed” or “strongly agreed” that “HPV vaccination should be offered to MSM in alternative settings such as GP practices or pharmacies” [23]. Disparities in knowledge and attitudes towards HPV vaccination for YMSM between SHCPs and GPs, as suggested in this study’s findings, may lead to differences in treatment and HPV prevention depending on where YMSM seek sexual health advice. Our findings indicate GPs may have a low level of knowledge regarding HPV vaccination among young MSM, and implementing a targeted HPV vaccination programme for YMSM prior to exposure to HPV to maximise the cancer prevention potential that involved GPs would need investment in clinician education, training, and support.

Studies in the United States of America explored reasons behind the low uptake of HPV vaccination for adolescent boys, where access varies on a state by state basis. In a national survey, Gilkey et al. found that paediatricians and family physicians delivered their recommendations for HPV vaccination in children inconsistently, sometimes not in a timely manner or with strong endorsement [19,25]. Alexander et al. also found variation in physicians’ recommendations of the HPV vaccine to young males, citing the “newness” and sexual nature of the vaccine as barriers [26]. The study authors suggest American family physicians do not feel they have the time or knowledge to counsel YMSM about the vaccine, and they do not believe they see them frequently. These findings are consistent with our results, providing further evidence of the need for extra support and training for GPs to help them identify YMSM and raise their awareness about the potential health benefits of HPV vaccination in this high-risk group.

This study utilised an adapted version of a validated survey instrument that has been delivered to SHCPs previously. There was minimal missing questionnaire data. Obtaining and comparing GP and SHCP knowledge, perceptions, and attitudes towards HPV vaccination for MSM (including young MSM) has proved insightful, given the JCVI recommendations that both settings could be used to deliver the vaccine. The lower levels of confidence and knowledge among GPs may help to explain the low uptake of HPV vaccination for MSM in the current pilot programme to date [27].

There are a number of limitations that be considered in the interpretation of this study’s findings. The cross-sectional design, convenience sampling approach, and exploratory nature of the study—using pre-determined survey statements—limits the ability to draw sound inferences about the reasons behind participant responses. The sample size is small, and while a response rate cannot be accurately calculated it is presumably quite poor considering the RCGP has over 50,000 members and BASHH has over 1000 members (some of whom are not based in the UK). There were no incentives offered for participation; a practice which is known to raise study participation rates in similar studies. Interviews with GPs who participated in this survey will provide more in-depth insight into their views and opinions regarding HPV vaccination for YMSM. 

## 5. Conclusions

GPs can potentially play a crucial role in the prevention of HPV-related diseases in YMSM. In order to implement the JCVI recommendation regarding HPV vaccination for MSM most effectively, YMSM should be identified early and offered the HPV vaccine with clear information. However, barriers to such implementation in primary care appear to still remain. If the findings of this exploratory work were confirmed in future research, interventions could be developed to raise awareness and educate GPs about the benefits of HPV vaccination for MSM, and to improve the skills of GPs in sensitively eliciting a patient’s sexual orientation to benefit the consultation and the patient–doctor relationship. There are also other potential settings for delivering HPV vaccination to YMSM to improve access, such as pharmacies and schools, which have not yet been explored.

## Figures and Tables

**Table 1:** Survey participant demographics.

Demographics		GPs (n = 38)	SHCPs (n = 49)	*p*
Age (mean years, SD)		39.84 (9.02)	41.02 (10.72)	0.59
Female gender (n, %)		26 (68.42%)	33 (67.35%)	0.46
Sexual orientation (n, %)	Heterosexual or straight	37 (97.37%)	38 (77.55%)	0.06
	Gay or lesbian	1 (2.63%)	9 (18.37%)	
	Bisexual	0	1 (2.04%)	
	Prefer not to say	0	1 (2.04%)	
Years of experience (mean, SD)		15.13 (9.10)	16.84 (11.56)	0.46

GPs: General Practitioners; SHCPs: sexual healthcare professionals.

**Table 2:** GPs and SHCPs knowledge regarding HPV vaccination.

Statement	GPs (n = 38)		SHCPs (n = 49)		Unadj OR	95% CI	Adj OR	95% CI
n (%)	High/Medium	Low/None	High/Medium	Low/None				
How would you rate your knowledge of HPV? *	34 (89.47%)	4 (10.53%)	47 (95.92%)	1 (2.04%)	0.19	0.02, 1.81	0.22	0.02, 2.44
How would you rate your knowledge of HPV vaccination for females? **	37 (97.39%)	1 (2.63%)	46 (93.88%)	1 (2.04%)	0.86	0.52, 14.24	1.77	0.05, 64.14
How would you rate your knowledge of HPV vaccination for MSM? ***	7 (18.42%)	30 (78.95%)	42 (85.71%)	6 (12.24%)	0.04	0.01, 0.12	0.02	0.00, 0.10
	**Yes**	**No**	**Yes**	**No**				
Are you aware of the recent JCVI recommendation for HPV and MSM?	6 (15.79%)	32 (84.21%)	41 (83.67%)	8 (16.33%)	0.04	0.01, 0.13	0.03	0.01, 0.11
Do you know enough about HPV and HPV vaccination to have an informed discussion with MSM patients?	10 (26.32%)	28 (73.68%)	44 (89.80%)	5 (10.20%)	0.04	0.01, 0.14	0.04	0.01, 0.14

* Missing responses from one SHCP; ** Missing responses from two SHCPs; *** Missing responses from one GP and one SHCP. Note—Hosmer-Lemeshow testing for goodness-of-fit *p* > 0.05; HPV: human papillomavirus; JCVI: Joint Committee on Vaccination and Immunisation; MSM: Men who have sex with me; Unadj OR: unadjusted odds ratios; Adj OR: adjusted odds ratios.

**Table 3:** GPs and SHCPs attitudes towards HPV vaccination for YMSM.

Statement	GPs (n = 38)		SHCPs (n = 49)		Unadj OR	95% CI	Adj OR	95% CI
n (%)	Yes	No/Unsure	Yes	No/Unsure				
HPV vaccination should be widely available for both sexes based on current evidence.	25 (65.79%)	13 (34.21%)	43 (87.76%)	6 (12.24%)	0.29	0.10, 0.86	0.30	0.09, 0.98
HPV vaccination should be widely available to MSM based on current evidence. ^	27 (71.05%)	11 (28.95%)	48 (97.96%)	1 (2.04%)	0.05	0.01, 0.45	0.30	0.09, 0.98
If there was a gender neutral HPV vaccination programme in the UK, would you still recommend targeted HPV vaccination?	16 (42.11%)	22 (57.89%)	34 (69.39%)	15 (30.61%)	0.32	0.13, 0.78	0.33	0.13, 0.88
Targeted HPV vaccination should be based on individual assessment of each MSM.	16 (42.11%)	22 (57.89%)	15 (30.61%)	34 (69.39%)	1.74	0.71, 4.30	1.91	0.70, 5.19
HPV causes too few cancers among MSM to make it worthwhile to offer vaccination. *	1 (2.63%)	36 (94.74%)	0	49 (100%)	N/A		N/A	
HPV causes too few cancer among HIV-positive MSM to make it worthwhile to offer vaccination. **	1 (2.63%)	36 (94.74%)	0	48 (97.83%)	N/A		N/A	
The majority of MSM would be willing to receive the HPV vaccine.	16 (42.11%)	22 (57.89%)	41 (83.67%)	8 (16.33%)	0.16	0.06, 0.44	0.13	0.04, 0.41
The majority of young MSM (<24 years) would be willing to receive the HPV vaccine.	21 (55.26%)	17 (44.74%)	41 (83.67%)	8 (16.33%)	0.28	0.10, 0.75	0.14	0.04, 0.49
HPV vaccination would encourage MSM to engage with sexual health services.	17 (44.74%)	21 (55.26%)	33 (67.35%)	16 (32.65)	0.45	0.19, 1.10	0.42	0.16, 1.11
Vaccinating MSM could increase the likelihood of unsafe sex.	3 (7.89%)	35 (92.11%)	1 (2.17%)	48 (97.83%)	3.97	0.40, 39.86	3.34	0.32, 35.02
I have the skills to identify MSM that would benefit from the HPV vaccine.	3 (7.89%)	35 (92.11%)	34 (69.39%)	15 (30.61%)	0.04	0.01, 0.16	0.03	0.01, 0.15
I am confident in recommending HPV vaccination for young MSM (<24 years).	12 (31.58%)	26 (68.42%)	43 (87.76%)	6 (12.24%)	0.06	0.02, 0.19	0.04	0.01, 0.18
I am confident that a young person would disclose their sexual orientation to me.	15 (39.47%)	23 (60.53%)	38 (77.55%)	11 (22.45%)	0.21	0.08, 0.55	0.17	0.06, 0.50

Unadj OR: unadjusted odds ratios; Adj OR: adjusted odds ratios; * Missing responses from one SHCP; ** Missing responses from one SHCP and one GP; N/A: unable to calculate OR. ^ Hosmer: Lemeshow goodness-of-fit test *p* < 0.05.

**Table 4:** Most frequently ranked factors preventing GPs and SHCPs vaccinating YMSM against HPV.

Factor	GPs Agree (n, %)	Rank	SHCPs Agree (n, %)	Rank
Lack of time in consultation	25 (65.79%)	1	12 (24.49%)	3
Do not see MSM frequently	20 (52.63%)	2	3 (6.12%)	5
Limited access to vaccine	14 (36.84%)	3	16 (32.65%)	2
Limited availability of vaccine	9 (23.68%)	5	34 (69.39%)	1

**Table 5:** Most frequently ranked factors supporting GPs and SHCPs vaccinating YMSM against HPV.

Factor	GPs Agree (n, %)	Rank	SHCPs Agree (n, %)	Rank
More time in consultation	30 (78.95%)	1	21 (42.86%)	1
Training about HPV vaccine	24 (63.16%)	2	14 (28.57%)	2
Computer prompts	21 (55.26%)	3	21 (42.86%)	1
